# Role of Topoisomerases in Pediatric High Grade Osteosarcomas: *TOP2A* Gene Is One of the Unique Molecular Biomarkers of Chemoresponse

**DOI:** 10.3390/cancers5020662

**Published:** 2013-06-04

**Authors:** Aurelia Nguyen, Christelle Lasthaus, Eric Guerin, Luc Marcellin, Erwan Pencreach, Marie-Pierre Gaub, Dominique Guenot, Natacha Entz-Werle

**Affiliations:** 1 Laboratoire de Biochimie et Biologie Moléculaire, CHRU Hautepierre, Avenue Molière, Strasbourg Cedex 67098, France; E-Mails: Aurelia.nguyen@chru-strasbourg.fr (A.N.); Lasthaus.christelle@chru-strasbourg.fr (C.L.); Eric.guerin@chru-strasbourg.fr (E.G.); Erwan.pencreach@chru-strasbourg.fr (E.P.); MariePierre.gaub@chru-strasbourg.fr (M.-P.G.); Dominique.guenot@inserm.fr (D.G.); 2 EA4438, Groupe Marqueurs Moléculaires de Progression Tumorale et de Sensibilisation aux Drogues Anti-Cancéreuses, University of Strasbourg, 3 Avenue Molière, Strasbourg 67000, France; 3 Laboratoire d’Anatomie Pathologique, CHRU Hautepierre, Avenue Molière, Strasbourg Cedex 67098, France; E-Mail: Luc.marcellin@chru-strasbourg.fr; 4 Service de Pédiatrie Onco-Hématologie, CHRU Hautepierre, Avenue Molière, Strasbourg Cedex 67098, France

**Keywords:** topoisomerase, osteosarcoma, chemoresponse

## Abstract

Currently, the treatment of pediatric high-grade osteosarcomas systematically includes one topoisomerase IIα inhibitor. This chemotherapy is usually adapted to the response to the neo-adjuvant therapy after surgery. The current and unique marker of chemoresponsiveness is the percentage of viable residual cells in the surgical resection. This late patient management marker has to be evaluated earlier in the therapeutic history of the patients on initial biopsy. Therefore, new biomarkers, especially those involved in the topoisomerase IIα inhibitor response might be good candidates. Therefore, our study was designed to target *TOP1*, *TOP2A* and *TOP2B* genes in 105 fresh-frozen diagnostic biopsies by allelotyping and real-time quantitative PCR. Our analyses in those pediatric osteosarcomas, homogeneously treated, highlighted the frequent involvement of topo-isomerase genes. The main and most important observation was the statistical link between the presence of *TOP2A* amplification and the good response to neo-adjuvant chemotherapy. Compared to adult cancers, the 17q21 amplicon, including *TOP2A* and *ERBB2* genes, seems to be differentially implicated in the osteosarcoma chemoresponsiveness. Surprisingly, there is no *ERBB2* gene co-amplification and the patients harboring *TOP2A* amplification tend to show a worse survival, so *TOP2A* analyses remain a preliminary, but a good molecular approach for the evaluation at diagnosis of pediatric osteosarcoma chemoresponsiveness.

## 1. Introduction

High-grade osteosarcoma is the most common and frequent form of pediatric bone cancer, observed mainly at adolescence and mostly localized in the long bones. The diagnosis of osteosarcoma is always confirmed histologically on the initial biopsy, done prior to any treatment. The current therapeutic management involves a preoperative chemotherapy, followed by a primitive complete surgical tumor resection and a post-operative chemotherapy. The post-operative treatment is adapted according to the histological grading system established by Huvos *et al*., witness of the response to the neo-adjuvant chemotherapy [1,2]. For several years, in the French Society of Pediatric Oncology (SFCE) recommendations, topoisomerase IIα inhibitors like etoposide and doxorubicin represent, along with high dose methotrexate and ifosfamide, the basis of therapeutic protocols [3]. The adjuvant treatment is adapted to the tumor chemoresponsiveness. In case of good responders (GR), the same topoisomerase IIα inhibitor is still recommended after the surgical resection, while in case of poor responders (PR), a switch is done to the alternative topoisomerase IIα inhibitor. Finally, the patients receive intravenous courses of cisplatin plus doxorubicin or ifosfamide plus etoposide. Surprisingly, some of the PR patients seemed to have a persistent sensitivity to topoisomerase IIα inhibitor, even after such a drug family was already administered as front line treatment. To date and to our best knowledge, this response to neo-adjuvant chemotherapy is the sole and persistent prognostic marker used widely in this bone cancer treatment. To our knowledge, no molecular biomarker(s) is (are) now used or will be used in a near future to predict before any treatment the response to neo-adjuvant chemotherapy, which could be, for example, intensified in case of a predictive poor response. Several research publications have focused on the worse prognosis, but could not highlight one or more surrogate markers useful at diagnosis to establish the potential response to chemotherapy [4]. Because of the wide use of topoisomerase IIα inhibitors in this bone cancer type during the neo-adjuvant treatment, one question remained: would the understanding of the topoisomerase IIα inhibitor efficacy and/or tumor resistance to this drug family help us to predict the GR or PR osteosarcoma patients at the initial biopsy? Large multicentric trials, especially in breast cancers, suggest that amplification or deletion of *TOP2A* gene may account for both sensitivity or resistance to topoisomerase IIα inhibitors [5]. First, to understand the role of *TOP2A* in the osteosarcoma chemoresponsiveness, *TOP2A* amplicon containing also *HER2/ERBB2* gene was studied in a homogeneously treated pediatric osteosarcoma tumor collection. To go further and understand the broader role of topoisomerases in osteosarcoma, we did not limit our study to topoisomerase IIα, but we extended the analyses to the three main types of DNA topoisomerases. These enzymes seem to play an essential and cooperative role in nuclear DNA topology and are expressed in normal osteoblasts where their presence is specific of proliferating cells [6,7]. Indeed, whereas the loss of normal osteoblast proliferation correlates with the downregulation of topoisomerase IIα expression, the isoform topoisomerase IIβ is upregulated when osteoblasts have plateaued in growth [6,8]. Other publications have also described the balance between topoisomerase I and II in the proliferation and induction of osteosarcoma cell migration [9]. Topoisomerase I was involved in osteoblast transformation [10]. Beside the potential role of topoisomerase IIα in drug sensitivity or resistance, the publications were then suggesting the interactive role of all topoisomerases in normal and malignant osteoblast proliferation and differentiation. To go further, *TOP1* and *TOP2B* genes were added to the analyses on diagnostic biopsies in a population of 105 pediatric patients, homogeneously treated with the OS94 protocol. *TOP1* gene is localized on chromosome 20q and the two topoisomerase II isoforms are encoded by the human *TOP2A* and *TOP2B* genes on chromosomes 17q and 3p, respectively [11]. To determine the status of these targeted loci, an allelotyping analysis was performed on biopsies’ DNAs and followed by a real-time semi-quantitative PCR targeting *TOP2A*, *TOP1* and *TOP2B* genes. The *ERBB2* gene study was implementing the status of 17q21 amplicon containing *TOP2A*. An immunohistochemical analysis helped us to understand the tumor protein expression of topoisomerase IIα combined with erbB2 and p53 immunohistochemistry. All results were correlated with histological responses to preoperative chemotherapy and patient outcomes. The statistical analyses had the purpose to investigate the relationship between the various genomic markers and the prognosis of our pediatric osteosarcoma cohort.

## 2. Results

This study was designed to analyze, first, the loci containing the topoisomerases and *ERBB2* genes. These genes might be implicated, as described in other adult cancers, in the treatment response of this homogeneously treated population of pediatric osteosarcomas (the patient characteristics are listed in Table 1). 

**Table 1:** Summary of patients’ characteristics.

**Number of patients**	105 patients
**Age**	12.9 years (median: 13 years, 4 to 18 years)
**Tumor sites**	81 tumors in the lower limbs (58 femurs); 14 tumors in the upper limbs (13 humerus); 10 tumors in other bone locations
**Chemoresponsiveness**	56 GR (53%)/49 PR (47%)
**Metastases at diagnosis**	18 patients (17%)
**Histological subtypes**	56 osteoblastic osteosarcomas; 10 fibroblastic osteosarcomas; 11 chondroblastic osteosarcomas; 28 patients with unknown data
**Overall Survival**	112 mo (median: 127 mo, 7 to 194 mo)
**Relapse Free Survival**	96 mo (median: 105 mo, 6 to 194 mo)
**Patients’ relapses**	41 patients
**Deceased patients**	29 patients

GR = good responder to neo-adjuvant chemotherapy, PR = poor responder to neo-adjuvant chemotherapy, mo = months.

Quantifying the target genes by real-time semi-quantitative PCR (QPCR) helped us to understand their deregulations, which could be linked to the chemotherapeutic responses. 

### 2.1. Frequent Allelotyping Rearrangements in 3p24, 17q21-q22 and 20q12-q13.1 Loci Were Correlated with the Gene Copy Variations of TOP1, TOP2A and TOP2B

In order to accurately evaluate each DNA region, two microsatellite markers were chosen to surround each gene in close vicinity. Therefore, microsatellite analysis was performed in all tumor specimens using six couples of primers targeting the 17q21 amplicon, containing *TOP2A* and *ERBB2* genes, and 3p24 and 20q12–q13.1 loci, containing respectively *TOP2B* and *TOP1* genes. By comparing biopsies’ DNAs and paired-blood DNAs, we frequently observed a change in the ratio between the two amplified alleles, defining the presence of an allelic imbalance (AI) in the tumor. No microsatellite instability (MSI), and only AI were detected, as already described in our previous publications [12,13,14]. The intensity of allelic ratio variation depends on the percentage of tumor cells in the biopsy specimens. The allelic ratio intensities ranged from 40% to 95%, and confirmed the high proportion of tumor cells in our collection, which was also evaluated by the histopathological analysis. The use of two microsatellites to surround each locus containing the genes improved the sensitivity of our technique. When both microsatellites were heterozygous, and thus informative for the allelotyping evaluation, only four cases for *D20S107* and *D20S855*, four cases for *D17S800* and *D17S1818* and two cases for *D3S1283* and *D3S700* showed discordant results between both microsatellites at the same locus. This rare proportion of discrepancies allowed us to consider patients as informative for these loci if they were heterozygous for both markers, or if at least one of them was altered or normal and the second one homozygous. 

Table 2 summarizes the results for each locus. Among these 105 patients, frequent rearrangements were detected in 66.5% of tumors in the 3p24 region, in 54% in the 17q12–21 amplicon and in 39.5% at the 20q11 locus, indicating that a deletion or an amplification of these regions are not rare events in pediatric osteosarcomas. 

Targeting directly the genes by QPCR allowed us to go further and see if our potential target genes were the ones involved in these frequent rearrangements. The QPCR confirmed the involvement of the *TOP1*, *TOP2A* and *TOP2B* genes. By allelotyping screening most of the rearranged samples were showing a deletion or an amplification of the topoisomerase genes. Few discordant results were observed. In only 12 patients, AI were not correlated with the paired gene deletion or amplification, but were concomitant with a normal gene status. These differences are explained by the lesser sensitivity of QPCR compared to microsatellite analyses. Surprisingly, *ERBB2* was only amplified in three patients and this amplification was concomitant with a *TOP2A* gene amplification. The topoisomerase genes were both amplified or deleted. The QPCR results are summarized in Table 3. Ninety two (92) cases were informative for *TOP1* analysis, 99 for *TOP2A* screening and 87 for *TOP2B* gene. *TOP1* was almost equally amplified in 12% (11/92) and deleted in 18.5% (17/92) of the biopsies, as well as *TOP2A* which was amplified in 21.2% of the biopsies (21/99) and deleted in 25.3% of tumors (24/99). The *TOP2B* gene seems to be differently rearranged, with a frequent deletion in 40.5% cases (35/87) and only 8% of amplified specimens (7/87).

**Table 2:** Microsatellite analyses in 105 pediatric high grade osteosarcomas (results expressed in patient numbers followed by the percentage, homozygous results are the non informative biopsies).

Locus targets	Allelotyping Results		
	Normal	Allelic imbalance (AI)	Homozygous result
*D20S107/D20S855* Combined results: 20q11 TOP1 locus 96 informative biopsies	58 = 60.5%	38 = 39.5%	9
*D17S800/D17S1818* Combined results: 17q21 *TOP2A*/*ERBB2* amplicon 87 informative biopsies	40 = 46%	47 = 54%	18
*D3S700/D3S1283*	26 = 33.5%	52 = 66.5%	27
Combined results: 3p24 *TOP2B* locus 78 informative biopsies			

**Table 3:** Real-time semi-quantitative PCR analysis (QPCR) focusing on *TOP1*, *TOP2A*, *ERBB2* and *TOP2B* genes (results expressed in patient numbers followed by the percentage).

Target genes	QPCR		
	Normal	Amplification	Deletion
*TOP1*	64 = 69.5%	11 = 12%	17 = 18.5%
*TOP2A*	53 = 53.5%	21 = 21.2%	25 = 25.3%
*ERBB2*	99 = 97%	3 = 3%	0
*TOP2B*	45 = 51.5%	7 = 8%	35 = 40.5%

### 2.2. A Significant Correlation Was Observed between TOP2A Rearrangements and Good Response to Neo-Adjuvant Chemotherapy

**Figure 1:**
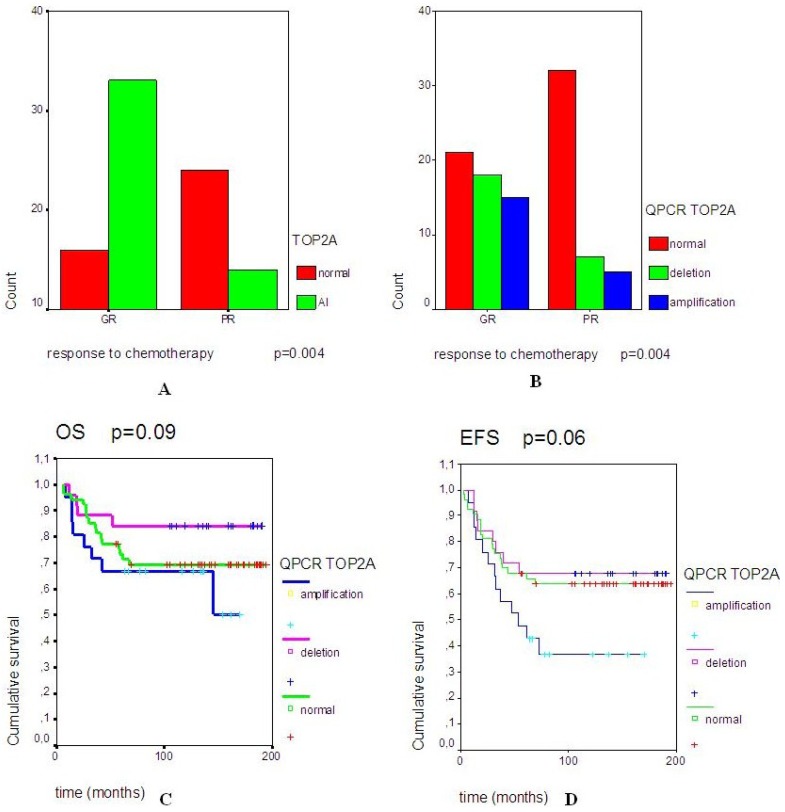
(**A**) shows the significant correlation (*p* = 0.004) between the presence of an allelic imbalance (AI) and the good responders to neo-adjuvant chemotherapy (GR). (**B**) focuses on the significant correlation (*p* = 0.004) between the chemoresponse [GR or poor response (PR)] and *TOP2A* gene status. (**C**) and (**D**) are representing the overall survival (OS) and event-free survival (EFS) curves in the 3 subgroups of patients, which are defined by a normal *TOP2A* gene, a *TOP2A* deletion or a *TOP2A* amplification.

Multiple chi2 test analyses were done computing the clinical data and DNA results. One of the significant (*p* = 0.004) correlations observed was between the good response to neo-adjuvant chemotherapy and the presence of *TOP2A* locus rearrangements (both deletion and amplification) (Figure 1A,B).

The amplification and the deletion of *TOP2A* gene seem to be equally involved in this chemosensitivity (Figure 1B). Surprisingly, the survival analyses showed a trend between a worse overall survival (OS) and the amplification of *TOP2A* gene (*p* = 0.09) (Figure 1C). Identically, a trend was observed between a worse event-free survival and the amplified *TOP2A* gene (*p* = 0.06) (Figure 1D).

**Figure 2:**
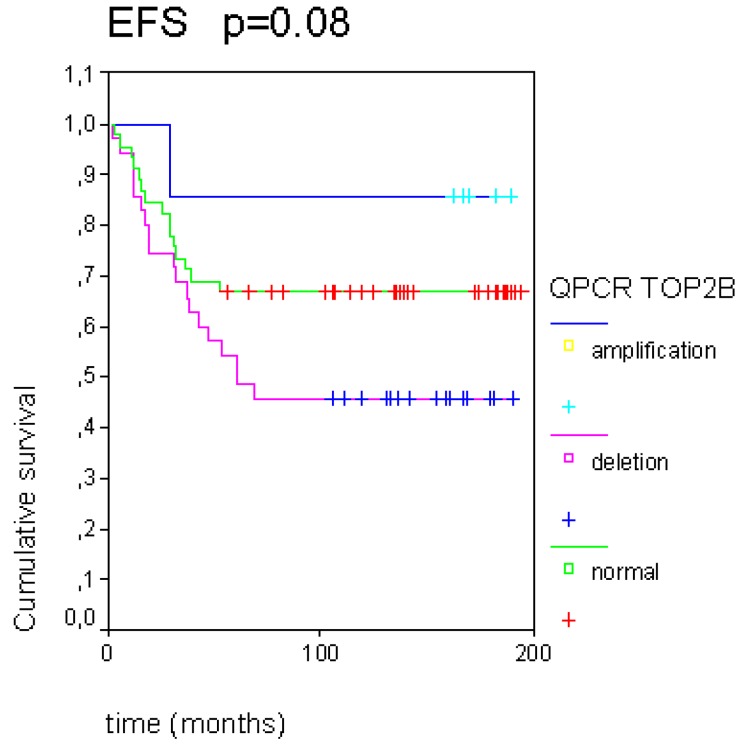
The *TOP2B* gene copy variations seem to have an impact on event-free survival (EFS) in this pediatric osteosarcoma population.

Another tendency was observed between a worse EFS and the deleted *TOP2B* population, whereas a better EFS was linked to the amplified *TOP2B* tumors (*p* = 0.08, Figure 2). 

**Figure 3:**
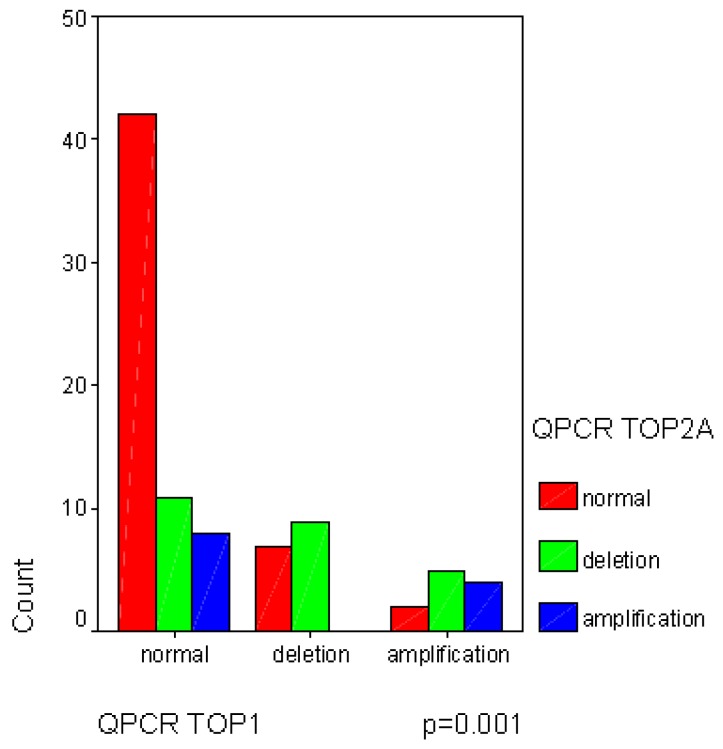
The significant correlation between *TOP1* and *TOP2B* gene copy variations.

No other statistical observation was noted, except the significant correlation between *TOP1* and *TOP2A* gene copy variations (*p* = 0.001), statistically linking the normal status of both genes (Figure 3).

### 2.3. Topoisomerase IIα Protein Expression and Gene Status

The immunohistochemical analysis, performed only in 17 patients, showed a strong and nuclear staining for topoisomerase IIα protein in almost all tumors (Table 4). The immunohistochemistry was only performed in few patients because of the absence of availability in tumor paraffin-embedded samples. 

**Table 4:** The results of topoisomerase IIα immunohistochemistry are showing a frequent link between a diffuse staining and an allelic imbalance at *TOP2A* locus (six patients). Among these six patients, four were presenting a *TOP2A* amplification (QCR analyses).

Patients	Topoisomerase IIα iummunohistochemistry				Allelotyping	QPCR
	Positive cells (%)	Staining intensity	Localization	Assessment		
Patient 1	10%	strong	nucleus	diffuse	homozygous	normal
Patient 25 *	50% (focal)	strong	nucleus	heterogeneous	normal	normal
Patient 26	10% (focal)	moderate	nucleus	heterogeneous	normal	normal
Patient 28	40–50%	strong	nucleus	diffuse	allelic imbalance	deletion
Patient 29	40% (focal)	strong	nucleus	heterogeneous	normal	normal
Patient 30	40% (focal)	strong	nucleus	heterogeneous	normal	normal
Patient 31	20%	strong	nucleus	diffuse	allelic imbalance	amplification
Patient 32	40% (focal)	strong	nucleus	heterogeneous	normal	normal
Patient 38	20%	strong	nucleus	diffuse	allelic imbalance	amplification
Patient 44	50% (focal)	moderate	nucleus	heterogeneous	normal	normal
Patient 47	30% (focal)	strong	nucleus	heterogeneous	normal	normal
Patient 55	50% (focal)	strong	nucleus	heterogeneous	homozygous	normal
Patient 64	50%	moderate	nucleus	diffuse	allelic imbalance	amplification
Patient 65	70%	strong	nucleus	diffuse	normal	normal
Patient 66*	30%	strong	nucleus	diffuse	allelic imbalance	amplification
Patient 67	60%	strong	nucleus	diffuse	allelic imbalance	amplification
Patient 68	50%	strong	nucleus	heterogeneous	allelic imbalance	deletion

**Figure 4:**
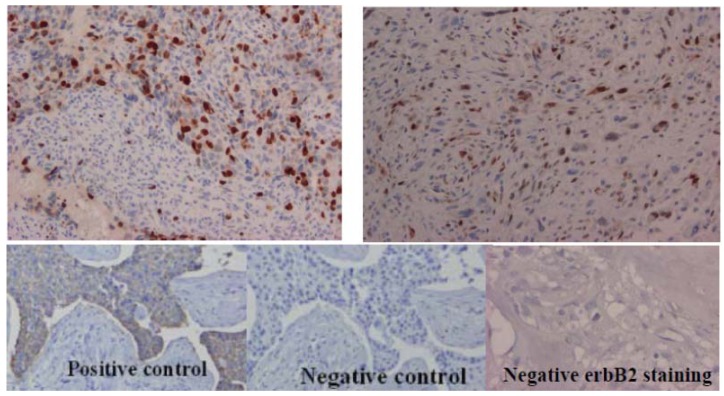
The immunohistochemical expression of topoisomerase IIα and ErbB2 proteins in pediatric osteosarcomas. (**A**) Heterogeneous topoisomerase IIα staining. (**B**) Diffuse topoisomerase IIα staining. (**C**) erbB2 protein staining.

The patient marked with * are those shown in Figure 4 for positive topoisomerase IIα immunohistochemical staining.

The osteosarcomas expressed differently topoisomerase IIα protein among the tumor sections. In nine patients, the protein expression was heterogeneously observed on the histological slides (Figure 4A) and was mapping the p53 protein staining in the same tumor (data not shown). The microsatellite analysis was normal in eight out of these nine patients, and one out of nine was presenting a deletion. In the eight remaining tumors out of 17, the topoisomerase IIα expression was diffuse in the tumor section (Figure 4B). 

For six patients, this diffuse staining was concomitant with the presence of an allelic imbalance in the 17q21 amplicon. Among these six rearranged tumors, five were presenting a *TOP2A* amplification. The topoisomerase IIα protein hyper-expression seems to be mainly linked to *TOP2A* gene amplification. No erbB2 expression was observed in these 17 tumors, confirming the QPCR analyses (Figure 4C). No correlation was observed between topoisomerase IIα protein expression and the patients’ clinical characteristics.

## 3. Discussion

Topoisomerase enzymes seem to be frequently involved in these pediatric high grade osteosarcomas with a high percentage of rearrangements ranging between 39.5% and 66.5%. To date and to our best knowledge, no previous publication has discussed such results on topoisomerases in a pediatric osteosarcoma cohort. The study results might be in accordance with the physiological role of topoisomerases in the normal osseous tissue, where topoisomerases are modulated to favor the proliferation, the growth and/or the migration of osteoblasts, so the high percentage of deleted *TOP2B* tumors seems to confirm the role of this enzyme, which is described by Feister *et al*. as downregulated during osteoblast growth [6,7]. Furthermore, the statistical trend between the worse EFS and *TOP2B* deletion population seems to link the induced malignant osteoblast growth to *TOP2B* downregulation, which is favoring the local osteosarcoma development. *TOP1* and *TOP2A* genes are frequently described with a balanced and linked regulation during osteoblast proliferation and migration [9,10]. The significant correlation between *TOP1* and *TOP2A* gene copy variations reinforced the molecular basis of *TOP1* and *TOP2A* linked expression in malignant osteoblasts and, consequently, in pediatric osteosarcomas. Our results also confirmed the predictive role of *TOP2A* gene in tumor chemo-sensitivity. In fact, the most important correlation we could identify was the significant link between the rearrangements of *TOP2A* locus and the good response to neo-adjuvant chemotherapy, known to include topoisomerase IIα inhibitors. Surprisingly, this correlation did not implicate *ERBB2* gene, as previously described in other cancers [15]. In fact, the *ERBB2* QPCR analysis mainly showed a normal gene status. Therefore, we could hypothesize that the 17q21 amplicon usually described in breast cancer [5,15,16,17,18] is not involved in the same way in pediatric osteosarcomas. Therefore, the status of *TOP2A* gene could be used as an early biomarker of chemoresponsiveness to neo-adjuvant treatment, but, is acting independently from the *ERBB2* gene. This absence of *ERBB2* amplification in pediatric osteosarcoma is concordant with previous publications in the field [19,20]. Furthermore, in pediatric osteosarcomas, the impact on survival of both *TOP2A* deletion and amplification seems to be different than those previously described in adult cancers [15,16]. The presence of *TOP2A* amplification tends to a slightly worse overall survival (Figure 1C,D; *p* = 0.08 and *p* = 0.06) compared to adult cancers. This observation allows us to hypothesize that the link between *TOP2A* status and sensitivity to chemotherapy is not the only biomarker involved in pediatric osteosarcoma chemosensitivity, prognosis and outcome, which are closely linked. In the literature, the chemosensitivity of pediatric osteosarcomas was also correlated to hypoxic markers, like HIF-1α [21], as well as Wnt-βcatenin pathway [22] or angiogenesis [23].

In conclusion, *TOP2A* gene status could be considered as a new diagnostic biomarker. It could be useful to analyze this gene in osteosarcoma tumors before any treatment. As in adult breast cancer, the next question will be to confirm these preliminary data on an extended cohort. To prove the independent prognostic impact of this molecular marker, an analysis has to be also performed in a osteosarcoma population treated differently. In fact, the chemoresponsiveness depends on the neo-adjuvant treatment. In osteosarcomas, any chemotherapeutic change is known to influence the frequency of patients with a good response to chemotherapy [24]. Therefore, multivariate and univariate analyses are needed to confirm TOP2A as a valuable biomarker.

The last question will be the type of molecular analysis to choose for this molecular diagnostic tool. As in other cancers [25,26], *TOP2A* gene status could be easily evaluated at a DNA level, where the quality controls and the independent reproducibility can be assessed, so a rapid and routinely done technique like FISH and/or QPCR could be performed for pediatric osteosarcomas.

## 4. Experimental Section

### 4.1. Tumor Banking and Patient Characteristics

A population of 105 pediatric primary high-grade osteosarcomas was recorded. Tumor tissues were collected from November 1994 to December 2005. The samples were obtained from the diagnostic biopsy of each patient. The molecular study was conducted in accordance with the Declaration of Helsinki. Clinical data were regularly updated. All population characteristics are summarized in Table 1. The 105 diagnostic biopsies were fresh-frozen and stored at −80 °C after the histological assessment by the pathologist. Control tissues were obtained from peripheral blood conserved on Whatman paper at room temperature. Paraffin-embedded sections were also obtained for 17 osteosarcomas.

### 4.2. DNA Extraction of Biopsy Samples

Tissues and blood paired DNA were purified as already described [14]. Tumor and blood genomic DNA concentrations were quantified by fluorometry, ranging from 50 to 400 ng/µL and from 1 to 10 ng/µL, respectively.

### 4.3. Microsatellite Analyses

Six microsatellites were analyzed on paired normal and biopsy DNA: *D20S107* and *D20S855* surrounding topoisomerase I gene (20q11), *D17S800* and *D17S1818* surrounding topoisomerase IIα gene (17q21) and *D3S1283* and *D3S700* surrounding topoisomerase IIβ gene (3p24) (see [27,28] for sequence primer description). DNA from both paired samples (10 ng) were amplified by PCR in a total volume of 25 µL using 0.125 µL of Taq polymerase and 4 pmol of both forward and Cy5 labeled reverse primers. PCR was carried out in an Omnigen Hybaid Thermocycler (Hybaid Ldt, Ashford, UK) using the following protocol: 5 min at 95 °C, 35 cycles of 1 min at 95 °C, 1 min at 55 °C and 1 min at 72 °C, followed by 5 min at 72 °C. The PCR products were analyzed by capillary electrophoresis on ABI PRISM^®^ Genetic Analyzer 3100 (Applied Biosystems, Foster City, CA, USA). The data were analyzed with the Genemapper Software (Applied Biosystems). This technique detects two types of rearrangements: a modification of the allele ratio in tumor DNA compared to paired blood DNA will be described as an allelic imbalance (AI) and the presence of additional peaks will be described as a microsatellite instability (MSI) [13,14]. The AI is the witness of deletion or amplification of the targeted locus, whereas the MSI is the witness of a mismatch repair defect, which was never observed in our population [12]. The intensity of the AI was directly proportional to the percentage of tumor cells and the AI was defined as the variation of the allele ratio between tumor and normal DNA. Then, an allelic variation above a cut-off of 20% is underlining the presence of a significant AI [12]. Each alteration was confirmed, at least, by a duplicate PCR.

### 4.4. Semi-Quantitative Real Time PCR (QPCR)

*TOP1* and *TOP2B* genes were quantified by QPCR using the SYBR Green I dye method (Roche, Diagnostics, Penzberg, Germany) and Light Cycler technology (Roche Diagnostics), whereas *TOP2A* was targeted using Taqman dye. Two internal control genes (*APP* and *DCK*, localized respectively at 4q11 and 21q21 loci) were used for the relative quantification in these population of osteosarcomas to overcome the high DNA rearrangements usually observed in this particular malignant bone tumor. All primers are listed in Table 5. *ERBB2* gene was quantified by QPCR using the Light Cycler (LC) HER2/neu DNA quantification commercial kit (Roche Diagnostics). The reference gene was an internal control localized in the centromeric region of chromosome 17. Both expressions were simultaneously detected by using specific pairs of hybridization probes. Each sample was analyzed in duplicate to confirm the results. 

**Table 5:** Primers for the semi-quantitative PCR of *TOP1*, *TOP2A* and *TOP2B* genes.

Genes	Forward primers	Reverse primers
	**Target genes**	
**TOP1**	5'-ATGGGTACAGTGTGCT-3' (intron 19)	5-'AGTTTGGAGGTTCCCAG-3' (exon 20)
**TOP2A**	5'-GCCATTGGCTGTGGTATTG-3' (exon 11)	5'-GAGAAGCTTCTCGAACATTGAG-3' (exon 12)
**TOP2B**	5'-GATTGGGTACTAGTACAGCT-3' (exon 16)	5'-GAATAGAAGGTAGGGGGATG-3' (intron 16)
	**Reference genes**	
**APP**	5'-TCAGGTTGACGCCGCTGT-3'	5'-ACCCCAGAGGAGCGCCACCTG-3'
**DCK**	5'-GCCGCCACAAGACTAAGGAAT-3'	5'-AGCTGCCCGTCTTTCTCAGCCAGC-3'

### 4.5. Immunohistochemical Analyses

The immunostains were performed using the avidin biotin peroxydase complex detection technique via an LSAB-2 kit (DAKO, Carpinteria, CA, USA). The anti-topoisomerase IIα monoclonal antibody topoIIα Ab-2, JH 2.7 (Neomarkers, Union City, CA, USA) was used as primary antibody (dilution 1/100). For ErbB2 protein, the standard rabbit polyclonal DA485 (HercepTest, Cytomation, Carpinteria, CA, USA) antibodies were used to analyze all specimens. The prepared paraffin-embedded sections were dewaxed, rehydrated and the endogenous peroxidase activity was blocked by H_2_O_2_/methanol. Then, a microwave antigen retrieval step was performed in 10 mM citrate buffer for 20 min. Thereafter, slides were sequentially incubated with the primary antibody, the biotinylated rabbit anti-mouse immunoglobulin and the streptavidin-biotin peroxidase complex (LSAB-2 kit, DAKO). Sites of peroxidase bound were visualized using diaminobenzidine (Dako, S3000), counterstained with Harris’s hematoxylin and mounted. The positive controls included in the experiment were defined as tissue previously shown to express topoisomerase IIα (colorectal tumor), whereas primary antibody was replaced by tris-buffered saline in the case of negative control. Immunostaining was graded according to the percentage of tumor cells presenting a positive stain, the staining intensity (weak, moderate or strong), the staining localization (nucleus or cytoplasm) and the staining assessment (diffuse or heterogeneous) (Table 4). These results were compared to the routinely done p53 immunohistochemistry in these bone tumors.

### 4.6. Statistics

Data were computed using SPSS 11.0 for Windows (SPSS, Inc., Chicago, IL, USA). The chi^2^ test was performed to analyze correlations between allelotyping rearrangements, QPCR results and clinical subgroups of patients. Survival function was estimated using the Kaplan Meier test.

## 5. Conclusions

These preliminary data to establish a molecular assessment at diagnosis of chemoresponse in pediatric osteosarcoma patients throughout the status of topoisomerase IIα at the gene and protein levels are promising, but questions remain and will be answered on an extended cohort of patients and independently from the current therapeutic protocol.
